# Human outbreak detection and best practice MPXV analysis and interpretation with squirrel

**DOI:** 10.1093/ve/veaf095

**Published:** 2025-12-10

**Authors:** Áine O’Toole, Eddy Kinganda-Lusamaki, Rachel Colquhoun, Connor Chato, Emily Scher, Chris Kent, Sam Wilkinson, Joshua Quick, Nicholas Loman, Ana T Duggan, Placide Mbala, Andrew Rambaut

**Affiliations:** Institute of Ecology & Evolution, University of Edinburgh, Edinburgh, EH93FL, United Kingdom; Institut National de Recherche Biomédicale, Kinshasa, P.O. Box 1197, Democratic Republic of the Congo; Service de Microbiologie, Département de Biologie Médicale, Cliniques Universitaires de Kinshasa, Université de Kinshasa, Kinshasa, P.O. Box 127, Democratic Republic of the Congo; TransVIHMI, Université de Montpellier, INSERM, IRD, Montpellier 34394, France; Institute of Ecology & Evolution, University of Edinburgh, Edinburgh, EH93FL, United Kingdom; National Microbiology Laboratory, Public Health Agency of Canada, Winnipeg, MB R3E 3M4, Canada; Institute of Ecology & Evolution, University of Edinburgh, Edinburgh, EH93FL, United Kingdom; Institute of Microbiology and Infection, University of Birmingham, B15 2TT, United Kingdom; Institute of Microbiology and Infection, University of Birmingham, B15 2TT, United Kingdom; Institute of Microbiology and Infection, University of Birmingham, B15 2TT, United Kingdom; Institute of Microbiology and Infection, University of Birmingham, B15 2TT, United Kingdom; National Microbiology Laboratory, Public Health Agency of Canada, Winnipeg, MB R3E 3M4, Canada; McMaster Ancient DNA Centre, Departments of Anthropology and Biochemistry, McMaster University, Hamilton, L8S 4L9, Canada; Institut National de Recherche Biomédicale, Kinshasa, P.O. Box 1197, Democratic Republic of the Congo; Service de Microbiologie, Département de Biologie Médicale, Cliniques Universitaires de Kinshasa, Université de Kinshasa, Kinshasa, P.O. Box 127, Democratic Republic of the Congo; Institute of Ecology & Evolution, University of Edinburgh, Edinburgh, EH93FL, United Kingdom

**Keywords:** MPXV, mpox, surveillance, software, phylogenetics, bioinformatics, APOBEC3, zoonosis, emerging disease, outbreak, genomic epidemiology

## Abstract

High numbers of reported mpox cases and recent identification of multiple sustained human outbreaks of mpox virus (MPXV) have highlighted the need for robust, best-practice genomic surveillance tools. In light of the severe acute respiratory syndrome coronavirus 2 (SARS-CoV-2) pandemic, many labs across the globe developed the capacity to do virus genome sequencing; however, MPXV presents additional analytical challenges due to its large genome size, tracts of low-complexity or repeat regions, genetically distinct clades, and the need to perform bespoke apolipoprotein B mRNA editing enzyme catalytic polypeptide-like 3 (APOBEC3)-mutation reconstruction. We present squirrel (Some Quick Reconstruction to Resolve Evolutionary Links), an open source bioinformatic tool that can perform clade-aware alignment, mutation quality assessment, phylogenetic inference, and automated APOBEC3-mutation classification on branches of the phylogeny. Squirrel can be run on the command line or launched through the EPI2ME graphical user interface through the squirrel-nf workflow, enabling robust analysis without need for the command line. With the interactive output report produced and publication-ready APOBEC3-reconstruction visualization, squirrel enables researchers to distinguish between zoonotic and sustained human outbreaks and help accurately inform public health responses.

## Introduction

**Figure 1:**
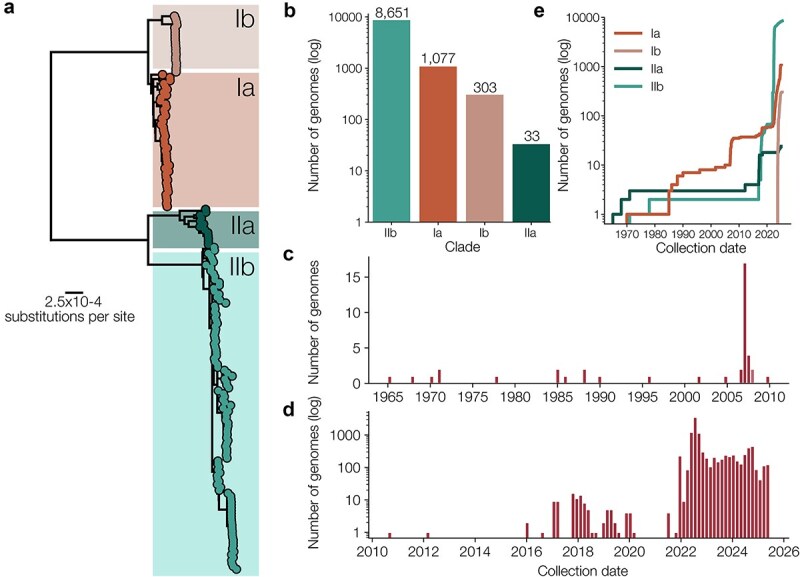
(a) The diversity of MPXV is currently split into four clades: Clades Ia, Ib, IIa, and IIb; (b) Clade IIb has the most genomes with 8 651 sequences available on Pathoplexus, and Clades Ia, Ib, and IIa have 1 077, 303, and 33 genomes respectively; (c) small numbers of samples were sequenced in the decades following MPXV discovery in 1958; (d) since the Clade IIb outbreak in 2017, many more genome sequences of the virus have been generated and shared online, with a total of 10 064 genomes on Pathoplexus that are sufficiently complete to assign clade; (e) Clade Ia has been sampled and sequenced over the last few decades; however Clade Ib is recently discovered with the emergence of the sustained human outbreak sh2023 that emerged from Sud Kivu province in the Democratic Republic of Congo.

MPXV, the virus that causes mpox, has historically been considered a zoonotic virus with limited capacity for sustained human-to-human transmission. However, our understanding of the epidemiology of mpox has shifted in recent years following a series of widespread, prolonged outbreaks in the human population. Whilst MPXV gained global attention in 2022 when it swept across the globe, evidence shows it had been spreading in the human population since at least 2016 (O’Toole et al. 2023). At the time of writing, there are two defined major clades of MPXV and three ongoing sustained human outbreaks of MPXV alongside the continued emergence of novel zoonotic cases of MPXV (Figure 1a).

A key tool in understanding the dynamics of MPXV emergence has been the identification of APOBEC3-driven mutations as a molecular signature of sustained transmission in the human population (Gigante et al. 2022, Isidro et al. 2022, O’Toole et al. 2023). *APOBEC3* refers to a family of host enzymes that bind single-stranded nucleic acids and deaminate cytosine residues to uracil. Through subsequent DNA synthesis, this change is observed as a C→T change in the viral genome (Pecori et al. 2022). Importantly, host-mediated APOBEC3 editing of the MPXV genome demonstrates a strong preference for TC dinucleotide motifs, resulting in TC→TT dinucleotide mutations (and the corresponding GA→AA mutations when editing occurs on the negative strand) (O’Toole et al. 2023). The genomes of humans and other primates encode seven paralogs of APOBEC3, whereas rodents possess only a single functional APOBEC3 gene (Conticello et al. 2004). Evidence suggests that APOBEC3F is likely the main driver of mutation of the MPXV genome (Suspène et al. 2023). This likely explains why signatures of APOBEC3 editing are only observed in MPXV genomes following sustained human transmission. This phenomenon enables researchers to infer whether the virus has been circulating in the human population and for how long, or whether the virus is likely caused by a recent zoonotic spillover. To answer these critical questions for new cases of mpox, public health labs and researchers have turned to genomic surveillance.

In the wake of the SARS-CoV-2 pandemic, many labs across the globe have developed the capacity for viral genomic sequencing, and this infrastructure and expertise can now be leveraged for MPXV genomic surveillance (Figure 1b-e). The availability of ARTIC-style tiled amplicon sequencing approaches for MPXV (Chen et al. 2023, Chris et al. 2024) has facilitated broader uptake, as it can be readily adopted by those familiar with the SARS-CoV-2 sequencing protocol. However, sequencing and analysis of MPXV present distinct challenges. MPXV is a large (~200 kb) double-stranded DNA virus with tracts of low-complexity and repetitive regions that can be problematic for some sequencing platforms. In addition, low complexity and repetitive regions may create difficulty during the genome assembly process, possibly incorporating uninformative or contaminant sequencing reads leading to single nucleotide polymorphisms (SNP) miscalls. As such, appropriate post-consensus quality control checks are critical for evaluating MPXV genomics. Moreover, MPXV has two genetically distinct clades and primer schemes optimized for one clade may perform poorly on the other. Mismatch between sequencing approach and analytical pipeline (e.g. metagenomic assembly pipeline for amplicon sequencing data) can result in low-quality genomes, incorrect SNP calls, and incorporation of primer artefacts. These errors can distort phylogenetic analysis, alter the interpretation of the data, and misinform public health responses.

In this article, we present squirrel (Some Quick Reconstruction to Resolve Evolutionary Links), which was developed in order to provide robust, reliable genome alignment and phylogenetics for MPXV, as well as providing automated APOBEC3-mutation reconstruction across the reconstructed phylogeny, enabling more accurate and rapid analysis of MPXV genomes.

## Materials and methods

### Availability and implementation

Squirrel is a command-line tool developed to provide best-practice analytic solutions for MPXV phylogenetics with global health capacity in mind. It does not require an internet connection to run, although it does require an internet connection to install for initial setup of the command line tool. It is Python-based with an embedded analysis pipeline built in a Snakemake framework (Köster and Rahmann 2018). All code is available on GitHub under an open-source GNU-GPL v3.0 licence at github.com/aineniamh/squirrel. Squirrel can be installed *via* bioconda, Docker, or directly from the GitHub repository. For users not comfortable with the command line, squirrel can be run using Oxford Nanopore’s EPI2ME GUI platform (labs.epi2me.io) through the ARTIC Network resource squirrel-nf (https://github.com/artic-network/squirrel-nf). Documentation describing installation, the full usage and configuration options, and an example data file is available at github.com/aineniamh/squirrel.

### Pipeline description

**Figure 2:**
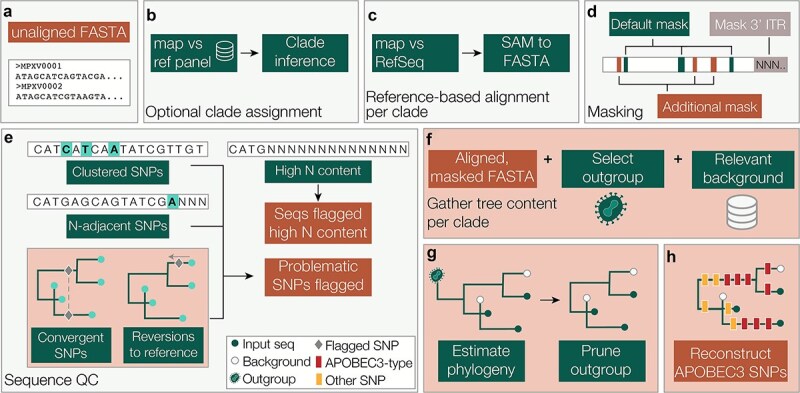
(a) The user can input an unaligned multi-FASTA file of MPXV genomes; (b) if ‘clade’ is set to ‘split’, the input genome sequences are assigned an MPXV clade using a custom random forest model trained on all MPXV data available on Pathoplexus; sequences will then be split by major clade for any downstream analysis; (c) the input sequences are mapped against the respective clade RefSeq genome and the output mapping file is converted to a FASTA alignment; (d) squirrel runs two kinds of masking on the input sequences; first, the 3′ inverted terminal repeat (ITR) region is converted to N bases; a set of problematic sites are also masked, which are known low-complexity or repetitive regions that many of the known sequencing issues surround; this mask file is relatively minimal and users may wish to supply an additional mask file to supplement the masking on genomes; (e) if squirrel is run in QC mode, it runs a module that generates a report flagging possibly problematic SNPs; SNPs flagged include clusters of SNPs, SNPs directly adjacent to an N, and also include convergent SNPs or SNPs that are reversions to the reference; this report is in the format that it can be used as the additional mask file in panel D; this file should be used to inform SNP investigation and confidence, and should not be used as a mask file without inspecting the SNPs themselves; (f) squirrel combines the input sequences with a clade-specific outgroup and optional background file; (g) IQ-TREE 2 is used to estimate a maximum likelihood tree (with ancestral state reconstruction if APOBEC3-reconstruction mode is active); (h) squirrel reconstructs which mutations occur along which branch in the phylogeny and calculates whether they are consistent with APOBEC3-editing or not; it produces an output report describing each mutation and the context around it, such as gene, codon position, amino acid mutation, dinucleotide context; squirrel produces an interactive HTML output report with the results summarized.

Squirrel combines various quality control and phylogenetic functionality into one user-friendly software package. A user can submit a FASTA file of unaligned MPXV genome sequences to squirrel (Fig. 2a). If the user specifies whether the ‘--clade’ is ‘cladei’ or ‘cladeii’, squirrel will select the respective RefSeq reference for either MPXV Clade I or II (Accession ID: NC_003310 or NC_063383). If the ‘--clade split’ flag is provided, squirrel will initiate the assignment pipeline that uses a custom MPXV random forest model to assign a clade to each input genome (Fig. 2b). This model is an adapted version of the pangolin lineage assignment random forest model, pangoLEARN (O’Toole et al. 2021). The inputs are then split by major clade (Clade I or II), and a separate alignment pipeline is initiated for each clade detected in the input. Squirrel can also run on variola virus (VARV) and if ‘variola’ is specified, the VARV RefSeq (Accession ID: NC_001611) is selected for alignment.

The alignment pipeline uses minimap2 to map all input sequences against the selected RefSeq reference genome and then gofasta (Jackson 2022) converts the output SAM file into a FASTA alignment (Fig. 2c). This approach maintains the reference genome length in the final multiple sequence alignment, which has the benefit that alignments of the same clade can easily be combined for additional analysis, but it does not permit insertions relative to the reference genome in the final alignment. The 3′ inverted terminal repeat region is masked out using Ns from the end of the genome (position 190 788 for MPXV or 184 722 for VARV) (Fig. 2d). A set of known repetitive and low-complexity sites that are problematic for sequencing and alignment are also masked out with Ns. The full set of sites is hosted on the squirrel GitHub at github.com/aineniamh/squirrel/blob/main/squirrel/data. Additional sites can be provided to squirrel with the ‘--additional-mask’ flag and those nucleotide positions specified will also be masked from the alignment across all sequences (Fig. 2d). Sites in specific sequences in the input FASTA file can be masked out by providing a ‘--sequence-mask’ csv file, and problematic sequences can be entirely skipped from the input file by providing an ‘--exclude’ csv file. The masking function is run by default but both the inverted terminal repeat (ITR) masking and the additional site masking can be toggled off.

When run with the optional ‘--seq-qc’ flag, squirrel will flag mutations that are directly adjacent to an N or a gap, a cluster of three or more mutations that occur within a 10-base window in the genome, and sequences with N content greater than 20%. Clusters of many SNPs in a small region of the genome can often be due to assembly or alignment errors. In a nucleotide sequence, N is often used to mask low-coverage regions, and a SNP directly adjacent to an N may be problematic. Similarly, a SNP next to a gap can be indicative of alignment issues and may require visual inspection of the alignment at that region. The user can then examine their data, decide whether the mutations or sequences are indeed problematic through visual inspection, and can then run a second round of squirrel and supply the output quality control (QC) files as ‘--additional-mask’ or ‘--exclude’ files in order to construct an alignment with these additional flagged mutations masked out (Fig. 2e).

**Table 1:** If not otherwise specified, squirrel will select an appropriate outgroup for phylogeneticanalysis based on the clade or virus specified.

**Virus**	**Clade**	**Outgroup accession**	**Alignment reference**
MPXV	I	KJ642615	NC_003310
MPXV	Ia	KJ642615	NC_003310
MPXV	Ib	KJ642613	NC_003310
MPXV	II	KJ642613	NC_063383
MPXV	IIa	KJ642613	NC_063383
MPXV	IIb	KJ642615	NC_063383
VARV	–	KJ642615	NC_001611

–, not applicable.

**Figure 3:**
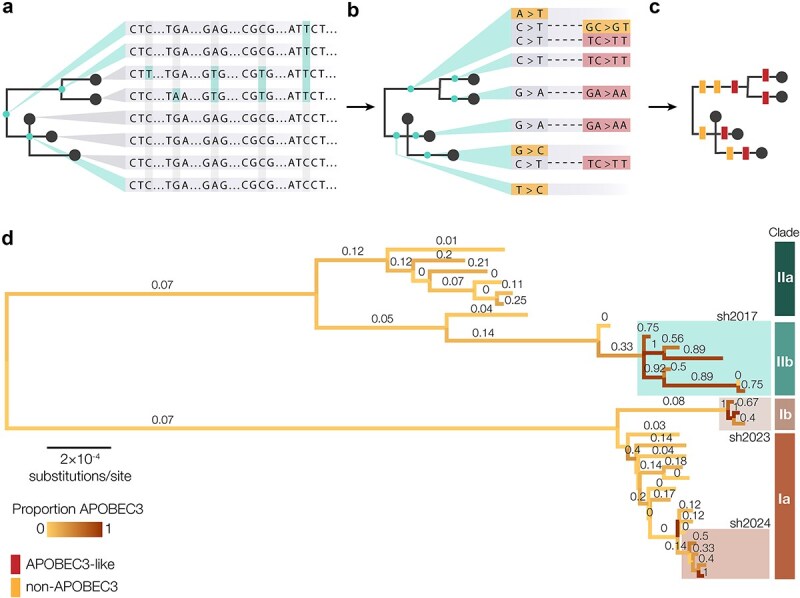
(a) Squirrel uses IQTREE2 to reconstruct the ancestral sequence states at each node in the phylogeny and catalogues a list of sites with unambiguous state changes; (b) squirrel maps which branches in the phylogeny each mutation occurs on and catalogues the mutations into whether or not they are consistent with APOBEC3-editing. C→T and G→A mutations—with either an upstream T or downstream A, respectively—are classed as mutations consistent with APOBEC3 editing; (c) these mutation classifications are output by squirrel in comma-separated value (CSV) form, alongside calculations of codon position and amino acid change reconstruction, in addition to graphically as a tree figure image with APOBEC3-like mutations shown in red and non-APOBEC3 mutations shown in yellow; (d) squirrel also produces an annotated tree file, with branches annotated with information on mutations that occur for along each branch and within each subtree below a given branch; here, branches were coloured by the proportion of APOBEC3-like mutations that occur along the branch using FigTree; the enrichment of APOBEC3-like mutations can be seen in the sustained human (sh) outbreaks sh2017, sh2023, and sh2024.

Optionally, the user can use squirrel to do a maximum likelihood phylogenetic analysis, including APOBEC3 mutation reconstruction on the phylogeny. Based on the clade specified or assigned, squirrel will select an appropriate outgroup to add to the alignment (described in Table 1 and Supplementary Fig. 1) to help correctly root the tree (Fig. 2f). For ease of analysis of new data, squirrel has the option to include a separate background file in addition to the input FASTA file. This allows the user to have a maintained reference file that can be combined with new data for analysis in the report. Squirrel also contains a minimal reference set that can be automatically added if it is run with the ‘--include-background’ flag. We use IQ-TREE 2 (Minh et al. 2020) to build a maximum likelihood tree with a Hasegawa-Kishino-Yano (HKY) substitution model (Hasegawa et al. 1985) and later prune the outgroup from the resulting phylogeny using jclusterfunk (https://github.com/rambaut/jclusterfunk) (Fig. 2g).

If APOBEC3 phylogenetics is selected, IQ-TREE 2 is used to reconstruct the ancestral genomes on the internal nodes of the tree (Figs 2h, and 3a). Squirrel catalogues the set of sites in the alignment with unambiguous state changes and identifies the state at internal nodes at those sites (Fig. 3a). Squirrel then calculates which changes occur on each branch in the phylogeny and maps the mutations to branches (Fig. 3b). Mutations that are neither C→T nor G→A are classified as non-APOBEC3 mutations. For C→T mutations, the position immediately upstream in the alignment is assessed for the appropriate dinucleotide context (T), and the position downstream of G→A mutations is assessed (A) (Fig. 3b). Based on this categorization, the mutations are mapped onto branches as either consistent with APOBEC3 editing or not (Fig. 3c). Squirrel uses the respective RefSeq reference GenBank file (Accession ID: NC_003310 or NC_063383) to calculate the position of each SNP within coding frames and categorizes the mutations by whether they are synonymous, non-synonymous, intergenic, or nonsense mutations and uses Grantham scores to predict how conservative or radial a given amino acid change is predicted to be. Another feature of squirrel, which is particularly useful when attempting to visualize large trees, is that it produces an annotated tree file with summary information on APOBEC3-like mutation counts, non-APOBEC3 mutation counts, the proportion of APOBEC3 mutations out of the total mutations occurring along each branch, and the binomial probability that a given subtree shows significant deviation different from the null expectation that 11% of observed mutations are APOBEC3-like by normal (i.e. non-host mediated) mutational processes (Fig. 3d).

**Figure 4:**
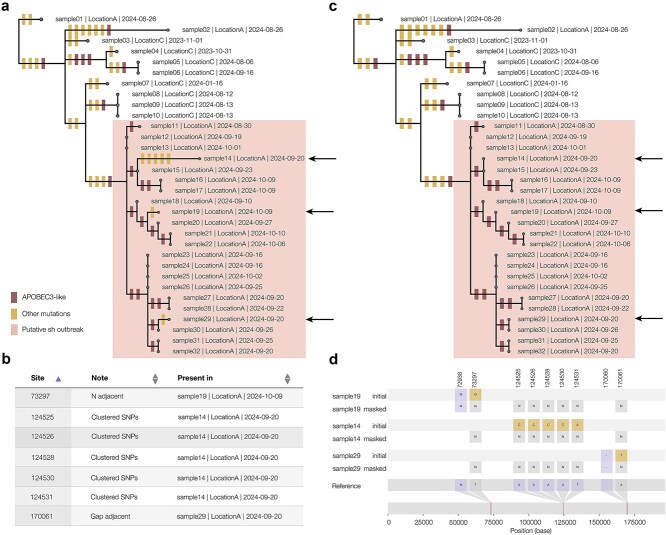
(a) The squirrel output report displays the phylogeny of input genome sequences with reconstructed mutations along branches coloured by whether they are APOBEC3-like (red) or not (yellow); in this example, a subset of the sequences appear to have an excess of APOBEC3-like mutations along the branches, shaded and labelled manually here as a putative sustained human (sh) outbreak; outside this clade, the branches show mutations more in line with the expected proportion of APOBEC3-mutations (~12%); within the putative outbreak clade, some sequences have non-APOBEC3 mutations (indicated by arrows), which is possible but would change our interpretation of the data; (b) running squirrel in QC mode produces an output table, displayed in the output report of sites that contain SNPs that may be due to bioinformatic error; SNPs are reported that lie directly adjacent to a gap or an N, and also if a high number of SNPs cluster together; (c) running squirrel again with the suggested mask file produces a report with the flagged SNPs masked out of the alignment; in this example case, the non-APOBEC3 mutations from the putative outbreak clade are masked out; (d) with the additional mask, each flagged column in the alignment is replaced with N bases.

We use the phylogeny and reconstructed mutations to create a graphical representation of the phylogeny with APOBEC3 mutations annotated onto the branches (Fig. 4) using Baltic (https://github.com/evogytis/baltic) and matplotlib. Mutations can be represented as coloured circles above the branches, or rectangles transecting the branches. APOBEC3-like mutations are shown in red and non-APOBEC3 mutations are shown in yellow. Squirrel can also produce an interactive tree figure with ape, ggtree, and plotly using the ‘--interactive-tree’ flag (Paradis and Schliep 2019, Yu 2020).

### Clade assignment model

We accessed all publicly available MPXV genome data with clade annotations on Pathoplexus.org and excluded any genomes that were <95% complete. The dataset included 7622 Clade IIb, 363 Clade Ia, 98 Clade Ib, and 33 Clade IIa genomes. We aligned all genomes against the Clade II RefSeq genome (Accession ID: NC_063383) using squirrel in QC mode, and subsequently re-aligned the genomes with the additional mask file produced by squirrel, which identified 5761 possibly problematic sites across the 8116 genomes. We adapted the pangoLEARN random forest model to accept clade annotations and trained a new random forest model on the cleaned 8116 genome dataset with clade annotations.

### Clade assignment tests

We compared three clade assignment methods for use within squirrel. First, we used the command line Nextclade v3.15.3 with the mpoxall-clades dataset (version 2025-04-25--12-24-24Z), which is the standard clade assigner for many labs doing MPXV genomics (Aksamentov et al. 2021). We also developed a pipeline whereby minimap2 v2.28-r1209 (Li 2018) is run (asm20) against a panel of curated background sequences representing each clade with only top hit recorded, and using the clade of the top hit as a clade assignment. Finally, we trained a custom random forest model on all available Pathoplexus MPXV data. This model was based on the pangoLEARN model used in pangolin for SARS-CoV-2 lineages (O’Toole et al. 2021).

In order to assess the various methods of clade assignment, we selected a representative background set of genomes from each clade (Accession IDs in Table 2). For each of the genomes in this dataset, we first tested how the approaches behave with increasing ambiguity by simulating a dataset of 31 000 genomes with incrementing levels of random ambiguity (N-bases) from 5% to 95%. We would expect that, as ambiguity increases, the accuracy will decrease due to missing SNP information. We also simulated genomes with incrementing genetic divergence from the known background panel to test how the assignment models behave with increasing distance from known diversity. To do this, we selected sites at random from across the MPXV genome at increasing percentage increments between 1% and 15% of sites and *in silico* mutated them away from the original nucleotide at that site. We repeated this random selection 100 times to generate the test dataset. We created an extended dataset by further simulating genomes with 15%–45% of sites randomly mutated in the same way, which represents far greater diversity than would be expected based on known MPXV diversity.

**Table 2:** Sequences from which data was simulated to test the assignment models. *record has since been removed from Genbank.

**Accession ID**	**Clade**
MT903346.1	Clade IIa
MT724769.1	Clade IIa
AY741551.1	Clade IIa
KP849470.1	Clade IIa
DQ011156.1	Clade IIa
KJ136820.1	Clade IIa
MN346698.1	Clade IIa
KJ642615.1	Clade IIb
KJ642617.1	Clade IIb
OP413718.1	Clade IIb
MK783027.1	Clade IIb
MK783033.1	Clade IIb
MN648051.1	Clade IIb
ON563414.3	Clade IIb
OP415257.1	Clade IIb
PP601220.1	Clade Ib
PP601212.1	Clade Ib
KJ642612.1	Clade Ia
KJ642619.1	Clade Ia
JX878412.1	Clade Ia
JX878413.1	Clade Ia
DQ011155.1	Clade Ia
MT724770.1^*^	Clade Ia
KJ642613.1	Clade Ia
PP601206.1	Clade Ia
JX878423.1	Clade Ia
DQ011154.1	Clade Ia

## Results

### Squirrel output report and QC functionality help ensure data inference is robust

The output report produced by squirrel includes links to the alignment and tree file, and also a graphical representation of the phylogeny with APOBEC3-mutation reconstruction displayed on the branches (Fig. 4a). If run in QC mode, the output report will contain a table listing the suggested sites to mask in another run and the rationale behind the masking (Fig. 4b). It is recommended to view these mutations in an alignment viewer (such as Geneious) to assess whether to mask these sites. Running squirrel again with the additional mask file will produce an alignment and phylogeny with the suggested columns masked out, which can help with interpretation of the data (Fig. 4c and d). In addition to MPXV, squirrel can also align and analyse VARV genomes. If run with VARV settings, squirrel will select the appropriate reference and mask file and produce a report showing the APOBEC3 reconstruction as with MPXV (Supplementary Fig. 2).

### Random forest model clade assignment is robust to ambiguity and diversity

**Figure 5:**
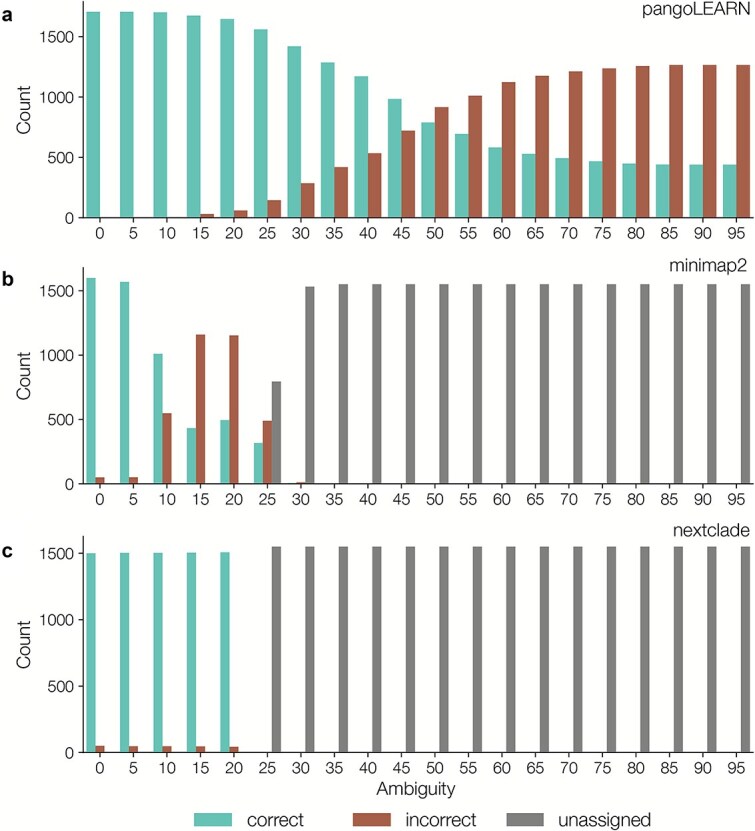
(a) We evaluated the MPXV random forest clade assignment model on simulated sequences with increasing ambiguity (*n* = 31 000); the model is robust to ambiguity, and genomes that were 90% complete or more were all correctly assigned a clade; the model’s ability to correctly assign a clade declines as the ambiguity increases and as such clade assignments for very incomplete genomes should be interpreted with caution; (b) as described in the minimap2 manual, it struggles to map beyond 20%, and the top hit becomes unreliable for clade assignment at greater than 10% ambiguity; (c) We tested the command line version of Nextclade against the simulated ambiguous data and the model cuts off at 25% ambiguity, but is highly accurate to that point.

**Figure 6:**
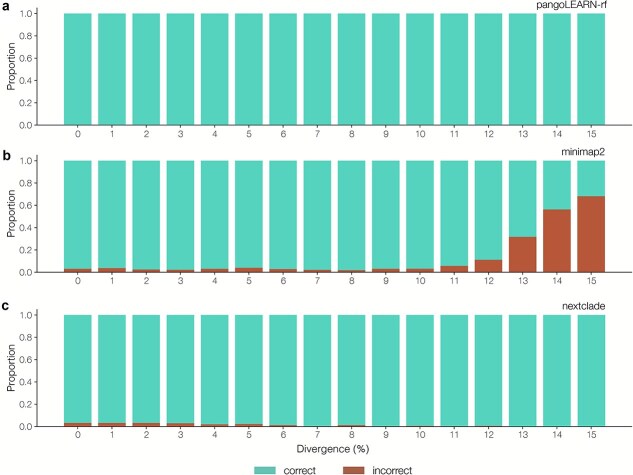
(a) We assessed the performance of the MPXV pangoLEARN random forest model against a simulated dataset of increasing divergence from known diversity, based on representative sequences from each of the four MPXV clades (*n* = 4 960); the pangoLEARN model was robust to divergence which is far beyond what we would observe in the MPXV population; (b) for minimap2, the accuracy began to decline as diversity increased, with more than 60% of assignments incorrect by 15% divergence; (c) Nextclade is robust to increasing divergence, and still accurately assigns clade at up to 15% divergence.

Clade assignment within squirrel was introduced in order to allow users to input data from a sequencing run with multiple clades, without needing the user to manually split the sequence file by clade, which could result in user error. We assessed a newly trained random forest model based on pangoLEARN, a custom pipeline involving minimap2 mapping against a reference panel and Nextclade parsimony placement on a mutation annotated tree. We tested the three methods against a dataset representing all four MPXV clades, with simulated ambiguity from 0% to 95% (*n* = 31 000). We found that pangoLEARN was most robust to ambiguity in the data, in comparison to minimap2 and Nextclade, which did not assign a clade beyond 25% ambiguity (Fig. 5). We also assessed the assignment approaches against a dataset of increasing SNP divergence from known MPXV diversity by simulating mutations on a set of representative genomes from 1% to 15% divergence from original sequence (*n* = 4960). We found that pangoLEARN and Nextclade were both robust to increasing simulated increasing diversity beyond known diversity; however, Nextclade required large memory requirements as the number of mutations increased (Fig. 6). We simulated an additional dataset up to 45% SNP divergence from known diversity, and even to this extreme distance, pangoLEARN manages to accurately assign 70.3% of sequences (Supplementary Fig. 3). The assignment approach using minimap2 was the least robust to data divergence from known diversity. For our purposes, we wanted a lightweight clade assignment method that was robust to both ambiguity and novel mutations and, based on our tests, the pangoLEARN random forest model was the most suitable.

## Discussion

The recent surge of MPXV cases, particularly those involving sustained human-to-human transmission, has underscored a need for reliable genomic surveillance tools capable of distinguishing zoonotic spillovers from sustained human outbreaks. Whilst the increased global capacity for genomic surveillance following the SARS-CoV-2 pandemic has facilitated broader adoption of genomic epidemiology for MPXV, inconsistent genome data quality has highlighted a need for a reliable analytical tool tailored to MPXV to help troubleshoot mutation calls, generate robust alignments, and perform APOBEC3-inference. Squirrel was developed to meet this need by providing an end-to-end solution for MPXV genomic alignment, phylogenetic inference, mutation quality control, and APOBEC3 mutation reconstruction and classification.

Within a nascent viral outbreak, genetic diversity among viral genomes is typically low, and phylogenetic reconstruction and inference are therefore informed by a limited set of mutations. Consequently, analysis can be sensitive to genome assembly or alignment errors. Careful evaluation of all mutations identified in alignments is essential for confident interpretation of the data. To support this process, squirrel can flag potentially suspect mutations in an alignment for further scrutiny. By default, squirrel masks out regions of the MPXV genome that are known to be challenging to assemble or align. Additional community resources provide complementary masking guidance, such as the mask file maintained by the International Pathogen Surveillance Network (hosted at github.com/WHO-Collaboratory/collaboratory-mpox-genomics-phylomasking). Importantly, we recommend that users do not automatically exclude all sites flagged by squirrel. Instead, they should be used to guide the user’s alignment curation process. Flagged mutations can signal upstream issues in the bioinformatics workflow, such as reference alleles being incorporated into the final consensus genome sequences, that merit further investigation and possible genome reassembly.

Squirrel supports both Clade I and Clade II alignments as well as analysis of VARV. It can assign an MPXV clade to input genomes using a custom random forest clade assignment model and then performs analysis on each clade with appropriately selected outgroups. In recent years, the number of sequenced MPXV genomes has increased substantially, revealing previously uncharted viral diversity. For example, genomic surveillance during the MPXV outbreak in eastern DRC revealed a novel subclade, Ib. It is likely that MPXV circulating in animal reservoirs harbours more, uncharacterized diversity. In addition, as the virus continues to circulate and evolve in the human population at the elevated APOBEC3-mediated rate, we will continue to see more diversity accumulate.

The random forest classifier for squirrel has been trained on currently known diversity, including the four clade categories. The model can be readily updated as new diversity is characterized; retraining and deployment can be carried out within a few hours. The update framework follows the same system developed for the SARS-CoV-2 lineage assignment tool, pangolin (O’Toole et al. 2021). The classifier is robust to increasing diversity, well beyond within-clade diversity; however, model accuracy declines with increasing ambiguity, as informative sites are missing. We therefore recommend a 20% ambiguity cut off for accurate clade assignment, which aligns with the cut off imposed in Nextclade. For genomes exceeding this level of ambiguity, we recommend using squirrel (or an equivalent tool) to run maximum likelihood phylogenetics and assess placement of the genome within a phylogeny against a background panel of genomes in order to further investigate clade assignments.

Squirrel has been designed to be an adaptable tool, allowing the user to incorporate a default background set for context and to provide a custom background set, making it as streamlined as possible to combine existing and newly generated genome data in an analysis. It is thus far the only tool available that can perform the APOBEC3 reconstruction, which is a critical analysis for informing public health measures around any nascent MPXV outbreak. Squirrel addresses a key analytical gap in MPXV genomic surveillance and the combination of its flexibility and ease of use makes it a valuable resource for both research and public health applications in the ongoing effort to monitor and control MPXV.

## Supplementary Material

squirrel_supplementary_veaf095

## Data Availability

All code and underlying data for squirrel are available on GitHub under an open-source GNU-GPL v3.0 licence at github.com/aineniamh/squirrel. Data used to generate figures were accessed from either GenBank or Pathoplexus and detailed in the article.
