# Supplementary material for: Human outbreak detection and best practice MPXV analysis and interpretation with squirrel
Source: Virus Evol. 2025 Dec 10;12(1):veaf095. doi: 10.1093/ve/veaf095 (PMC12822064; doi:10.1093/ve/veaf095)
Supplement: squirrel_supplementary_veaf095 [file squirrel_supplementary_veaf095.pdf]

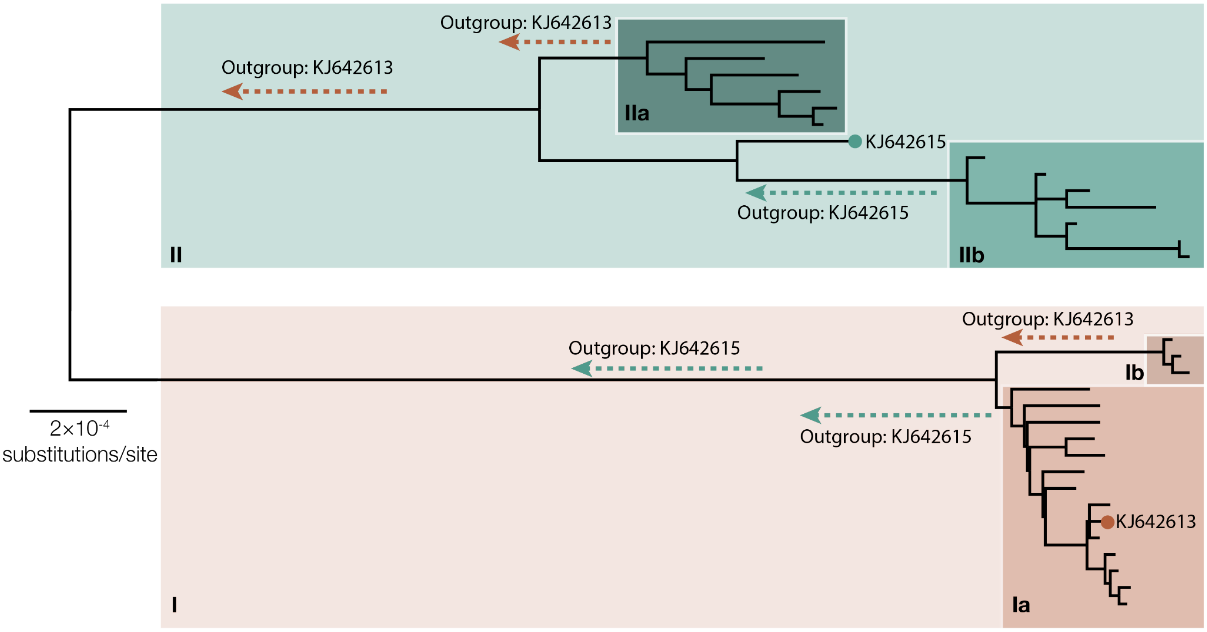

Supplementary Figure 1 | Outgroup selection for each of the clades within squirrel attempts to account for the potential for more basal clade diversity that has not yet been sampled and sequenced. For Clade I and Clade II overall, an outgroup from the opposite major clade is selected. Within each clade, the most basal subclade (i.e. Ia and IIa) also has an outgroup selected from the opposite major clade. It would also likely be appropriate to select a custom outgroup within a given major clade in this instance. For the least basal subclades (i.e. Ib and IIb), an outgroup within the respective major clade is selected. Arrows indicating outgroup are coloured by which clade the outgroup belongs to (green for Clade II, orange for clade I).

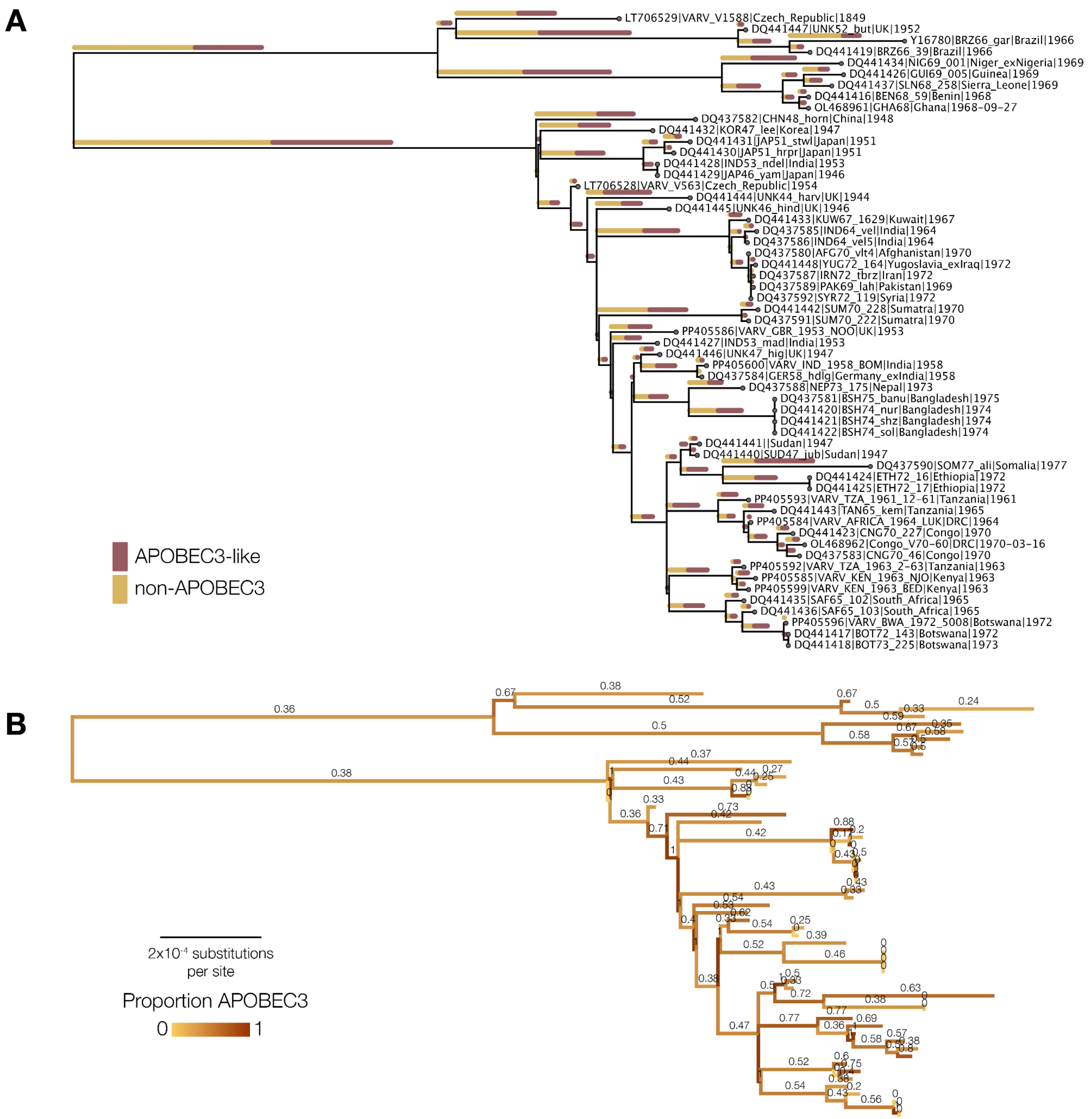

Supplementary Figure 2 | A) Squirrel can process and perform APOBEC3 reconstruction on variola virus (VARV) genomes. Here we ran squirrel in APOBEC3-reconstruction mode on 57 open-access modern VARV genomes sourced from Genbank (accessions shown in figure). B) This reconstruction will also produce an annotated tree file that can be viewed in FigTree, displaying APOBEC3 proportion on each branch.

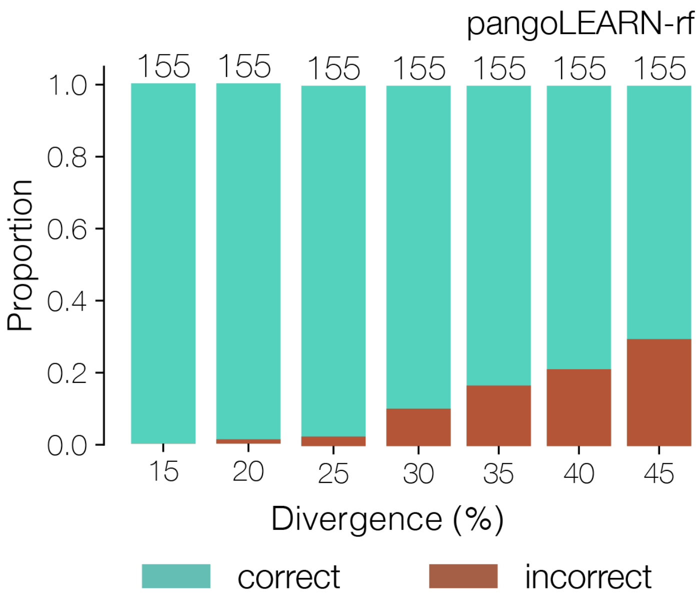

Supplementary Figure 3 | We simulated a dataset of up to 45% divergence from known MPXV diversity and found the pangoLEARN random forest model for MPXV clade assignment was robust to this diversity and still accurately assigned 70.3% of sequences at 45% divergence from known MPXV, which far exceeds known diversity within the species.
